# Dribble deficit as an effective measure of dribbling ability independent of sprinting performance in professional female handball players

**DOI:** 10.3389/fphys.2024.1506893

**Published:** 2025-01-10

**Authors:** Ljubomir Pavlović, Anja Lazić, Nedim Čović, Rado Pišot, Milan Petronijević, Zoran Milanović

**Affiliations:** ^1^ Faculty of Sport and Physical Education, University of Niš, Niš, Serbia; ^2^ Science and Research Centre of Koper, Koper, Slovenia; ^3^ Faculty of Sport and Physical Education, University of Sarajevo, Sarajevo, Bosnia and Herzegovina; ^4^ Faculty of Sport and Physical Education, University of Belgrade, Belgrade, Serbia; ^5^ Faculty of Sports Studies, Incubator of Kinanthropological Research, Masaryk University, Brno, Czechia

**Keywords:** dribbling, technical proficiency, team handball, agility, movement

## Abstract

**Purpose:**

The purpose of this study was to determine the relationship between linear and change-of-direction sprinting performance with dribbling performance and Dribble Deficit in professional female handball players.

**Methods:**

Eleven professional female handball players (mean age: 21.12 ± 4.34 years; body height: 171.59 ± 4.52 cm; body weight: 66.29 ± 5.73 kg) participated in the study. Each participant completed several linear (sprint over 10, 20, and 30 m) and change-of-direction tests (slalom test, zig-zag test, 505 test), first without the ball (sprinting performance) followed by ball dribbling (dribbling performance). Dribble Deficit was calculated indirectly as the time difference between the best trial while dribbling minus the best trial without dribbling.

**Results:**

A large to very large correlation was observed between the linear sprint and dribbling performance (r = 0.53–0.78), as well as between change-of-direction sprinting performance and dribbling performance (r = 0.66–0.88). The study also showed a moderate to perfect relationship between linear dribbling performance and Dribble Deficit (r = 0.46–0.93), and a large relationship between change-of-direction dribbling performance and Dribble Deficit (r = 0.54–0.55), while the relationships between linear sprinting performance and Dribble Deficit (r = −0.51–0.21) and between change-of-direction sprinting performance and Dribble Deficit (r = −0.14–0.26) were small and non-significant.

**Conclusion:**

In summary, Dribble Deficit reflects dribbling ability independent of sprinting ability and refines its application for practical use in assessing dribbling skills in female handball players.

## Introduction

Team handball is characterized by frequent intensity fluctuations, that require players to coordinate various physical demands, including side cutting, tackles, running, sprinting, jumping, and change-of-direction (Ingebrigtsen et al., 2013; Michalsik et al., 2014). These physical demands must be integrated with technical skills such as ball throwing, catching, passing, and dribbling (Michalsik et al., 2015; Stamenković et al., 2023). Dribbling demands during handball gameplay are relatively low compared to other team sports (Stamenković et al., 2023) but dribbling skills and dribbling performance time have been identified as critical attributes for handball players. These technical skills assist players in maintaining field position and ball possession by evading tackles and opponents’ attempts to intercept (Karcher and Buchheit, 2014). Dribbling is predominantly dependent and executed in conjunction with fast running and sprinting or intense change-of-direction during low to high-speed transitions or in one-on-one duels (Ingebrigtsen et al., 2013; Karcher and Buchheit, 2014; Stamenković et al., 2023). In addition, dribbling performance time proficiency is widely used as a criterion for identifying talent in handball (Lidor et al., 2005; Serrien and Baeyens, 2018), therefore, effective measurement methods are necessary to accurately identify talented players.

Recently, composite handball game-based tests have been used to evaluate specific physical and technical performance, including sprinting performance with and without the ball (Schwesig et al., 2016; Wagner et al., 2016; Michalsik et al., 2021; Wagner et al., 2022). However, these tests were often impractical and limited (Wagner and Hinz, 2023) as dribbling (independent of sprinting ability) was not included as a component of the tests, nor considered in the prediction of handball performance (Almeida et al., 2020; Bojić et al., 2020). Currently, dribbling assessment in handball is typically performed via field tests that incorporate straight-line or multidirectional running. Furthermore, dribbling quality and efficiency are determined traditionally by measuring the time required to complete the course while dribbling the ball (Lidor et al., 2005; Manchado et al., 2013; Schwesig et al., 2016). However, dribbling speed in those assessments is largely determined by a player’s running and sprinting speed (Scanlan et al., 2018a; Wilson et al., 2020), so the protocols that only include dribbling, running, and sprinting speed are questionable in assessing dribbling performance despite already confirmed validity. Therefore, traditional methods have often been confounded by an athlete’s sprinting abilities, failing to provide a true representation of dribbling performance (Stamenković et al., 2023). However, understanding the difference between sprinting and dribbling speed performance (both in straight line and change-of-direction scenarios) can contribute significantly to improving dribbling quality and game performance.

To address the limitation of traditional dribbling assessments, which are often confounded by sprinting ability, Scanlan and colleagues (Scanlan et al., 2018b) introduced a novel measure of dribbling performance time called Dribble Deficit. Briefly, Dribble Deficit is the additional time taken to complete sprinting assessments while dribbling compared to sprinting without a ball. Notably, Dribble Deficit has been proposed as a more reliable and valid metric to quantify dribbling performance, independent of confounding factors such as acceleration and technical proficiency (Scanlan et al., 2018a; Ramirez Campillo et al., 2021a). These findings are further supported by the efficacy of the Dribble Deficit as a more precise measure of asymmetry in dribbling skills compared to traditional assessment methods, highlighting its potential to identify imbalances between the dominant and non-dominant sides (Trecroci et al., 2020). Specifically, recent studies (Scanlan et al., 2018b; Conte et al., 2020; Ramirez-Campillo et al., 2021b) reported moderate to very large significant relationships between linear sprinting and dribbling performance (r = 0.64–0.82) and change-of-direction sprinting and dribbling performance (r = 0.44–0.88) in semi-professional, junior and collegiate male basketball players. However, the same studies described the relationship of Dribble Deficit with linear and change-of-direction sprint times as non-significant trivial, small, or moderate (r = −0.45 to 0.22). Similarly, Stamenković et al. (2023) found interdependence between sprinting and dribbling performance using the traditional and Dribble Deficit approach among professional male handball players. Furthermore, the aforementioned male handball study indicated that both linear sprinting and change-of-direction performance were significantly associated with dribbling performance (r = 0.65–0.76). In contrast, a trivial to small, non-significant relationship was observed between linear sprint and change-of-direction performance and Dribble Deficit (r = 0.14–0.35) (Stamenković et al., 2023). The authors additionally concluded that further research on Dribble Deficit is needed, particularly in female handball players, to determine whether Dribble Deficit is also independent of players’ physical performance because of the lack of research performed on elite female athletes, limiting the development of evidence-based approaches to practice (Emmonds et al., 2019).

Unsurprisingly, evidence-based knowledge for coaches and sports scientists regarding elite female handball is limited (Manchado et al., 2013) and more importantly, the translation of results from studies on male to female athletes may be erroneous (Emmonds et al., 2019). Within the area of investigation, research on Dribbling Deficit (Scanlan et al., 2018b; Conte et al., 2020; Ramirez-Campillo et al., 2021a; Stamenković et al., 2023) has primarily focused on male athletes (e.g., collegiate and semi-professional basketball players, football players, and handball players), while the application of Dribbling Deficit in female athletes is largely overlooked. The lack of information on the contribution of dribbling and its relationships to sprint performance are critical gaps that need to be addressed. Notably, biological factors such as muscle mass, tendon and ligament strength (Mendiguchia et al., 2011), and hormonal fluctuations during the menstrual cycle (Emmonds et al., 2019; Janse et al., 2019; Meignié et al., 2021) may influence skill execution, including dribbling performance. Furthermore, specific running sprint performance threshold, especially sprinting performance, is lower in elite female handball players compared to males (Michalsik et al., 2013), while dribbling performances are similar. Therefore, there is a need to validate the new Dribble Deficit method in female handball players. Thus, to the best of our knowledge, no study using the Dribble Deficit approach has been conducted in women’s handball or other female team sports, so the results considering gender-related differences are also unexplored.

Therefore, the purpose of this study was to determine the relationship between linear and change-of-direction sprinting performance with dribbling performance and Dribble Deficit in professional female handball players. We hypothesized that there would be a higher amount of shared variance between sprinting performance and dribbling performance while sprinting performance and Dribble Deficit would have a lower variance in both linear and change-of-direction patterns.

## Methods

### Participants

Eleven professional female handball players (mean age: 21.12 ± 4.34 years; body height: 171.59 ± 4.52 cm; body mass: 66.29 ± 5.73 kg) competing in the Women’s Handball Super League participated in this study. To determine the required sample size, an *a priori* power analysis using G*Power software (G*Power, Version 3.1, Heinrich-Heine University Düsseldorf, Germany) was used. Relying on previous similar Dribbling Deficit studies in basketball and handball (Scanlan et al., 2018a; Stamenković et al., 2023), it was determined that a sample size of 10 participants would be a sufficient number to meet the criteria (alpha = 0.05; beta = 0.80; coefficient of determination = 0.5). The inclusion criteria were as follows: (1) female handball players playing the first National Division; (2) had at least 3 years of structured handball training; (3) frequency of structured handball training at least 3 times per week; (4) without any chronic diseases, injuries and contraindications to participate in the study. Moreover, we did not restrict the age range, considering it is widespread for first National Division female handball players to be younger than 18 years old. Prior to their assessments, all players were screened by team doctors and physiotherapists for injuries and medical conditions to ensure no health contraindications prevented safe participation. All participants were members of the same handball team. Moreover, the study was conducted at the end of the competitive season, during which the participants completed 5 days of structured handball training (each lasting 90 min) without any competitive matches taking place during the study. All participants were free from injuries and medical conditions that could affect their ability to participate in the study safely. The specific aims, benefits, risks, safety measures, and procedures were explained both verbally and in writing to all participants before all players or their guardians, if under 18 years of age, gave their written consent to participate. The procedures were approved by an institutional Human Research Ethics Committee.

### Procedures

This study employed a within-subject, cross-sectional design with all tests done in one session. Before testing, all participants underwent a standardized 15-minute warm-up consisting of moderate-intensity jogging (5 min) with dynamic stretching exercises (5 min) progressive sprints, and specific handball drills, including ball handling maneuvers (5 min). Immediately, following the warm-up, tests were conducted in the following order: (1) 30 – m linear sprints; (2) slalom test; (3) zig-zag test; (4) 505 test. All tests were performed twice, first without the ball and then while dribbling, with a 3-min passive rest between each attempt. The fastest trial from each participant, recorded in seconds, was used for further statistical analysis. Standardized competition handballs (Molten brand, HX5001-BW model, size 2) were used for the tests where the ball was required. Performance times on both linear sprint performance and change-of-direction tests were assessed using the Witty photocell system (Witty System, Microgate, Bolzano, Italy). According to previous guidelines (Nimphius et al., 2016) timing gates were positioned 1.2 m above the ground with 1.5 m apart, and 0.3 m behind the starting line to avoid participants prematurely breaking the signal and/or their arms passing through the gate first. Participants were familiar with all tests since these tests are part of the usual fitness assessment battery. Additionally, when performing the tests, participants were instructed to always have their dominant foot on the starting line at the beginning of the test, while in change-of-direction tests with the ball, participants always initiated movement with the dominant hand but were required to switch the ball from the dominant to non-dominant hand during the tests. Testing took place on a hardwood indoor handball court in the morning hours, from 9.00 to 11.00 A.M., coinciding with the typical morning training schedule. Participants were advised to refrain from alcohol, caffeine, or other stimulants 24 h prior to testing and to avoid strenuous exercise.

### Linear sprint performance

Linear sprint performance was evaluated by instructing participants to perform maximum-effort sprints over a distance of 30 m from a standing start, with split times recorded at 10 m, 20 m, and 30 m in order to measure both acceleration and maximal linear sprint performance. The sprint performance was determined using four pairs of photocell timing gates placed at the starting line and at 10 m, 20 m, and 30 m. Participants were instructed to run at maximum capacity through all gates without stopping. The timer was automatically activated as participants crossed the first gate at the starting line with split times at 10 m, 20 m, and 30 m. This test is widely utilized to assess linear sprint performance in handball players (Stamenković et al., 2023).

### Change-of-direction testing

#### Slalom test

**FIGURE 1:**
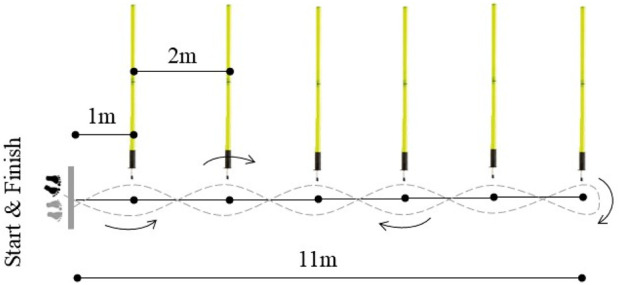
Layout of the slalom test performed with and without dribbling.

The slalom test setup consisted of 6 cones placed at a distance of 2 m between each other with the first cone positioned 1 m from the starting line. Participants began from a standing position and maneuvered around the cones in a semi-circular running pattern, alternating sides with each cone. They were allowed to choose their initial side (left or right) for the first cone. Subsequent cones required them to change direction by passing on the opposite side used for the previous cone. Around the last cone, participants executed a 180° turn and returned to the starting line using the same slalom pattern passing through timing gates (Figure 1). Participants were advised to avoid wide turns during the test. The test was repeated if participants did not perform the test correctly or lost control of the ball. The Slalom test is widely used for assessing change-of-direction performance in handball players (Matthys et al., 2013; Saavedra et al., 2020; Stamenković et al., 2023), and has been shown to be reliable (ICC: 0.992; CV: 2.9) (Sporis et al., 2010).

#### Zig-Zag test

**FIGURE 2:**
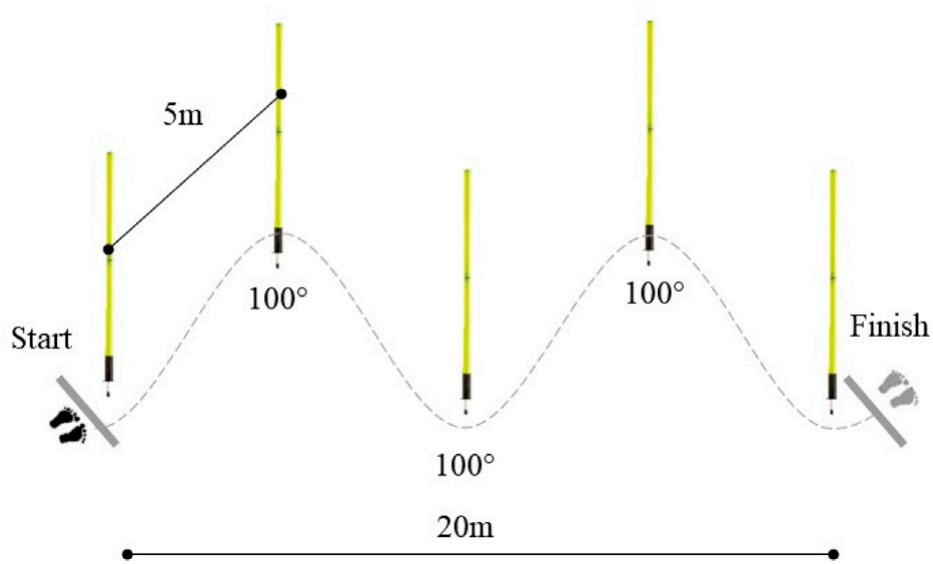
Layout of the zig-zag test performed with and without dribbling.

The zig-zag test was used to assess change-of-direction performance according to the previously described procedure (Pereira et al., 2018). Briefly, the test consisted of four cones placed at 5-m apart, covering a total distance of 20-m. The cones were positioned at 100° angles, allowing the participants to perform rapid acceleration and deceleration movements around each cone (Figure 2). Participants were instructed to perform the test as fast as possible and not to slow down before the last set of timing gates. The test was stopped and repeated if: 1) participants bypassed (cut) the cone on the inside, 2) the ball fell out of their hands, or 3) there was no hand change during dribbling. Moreover, the zig-zag test has been already used in handball players (Pavlović et al., 2018) demonstrating high reliability (ICC 0.89; CV: 0.05) (Sekulic et al., 2013).

#### 505 test

**FIGURE 3:**
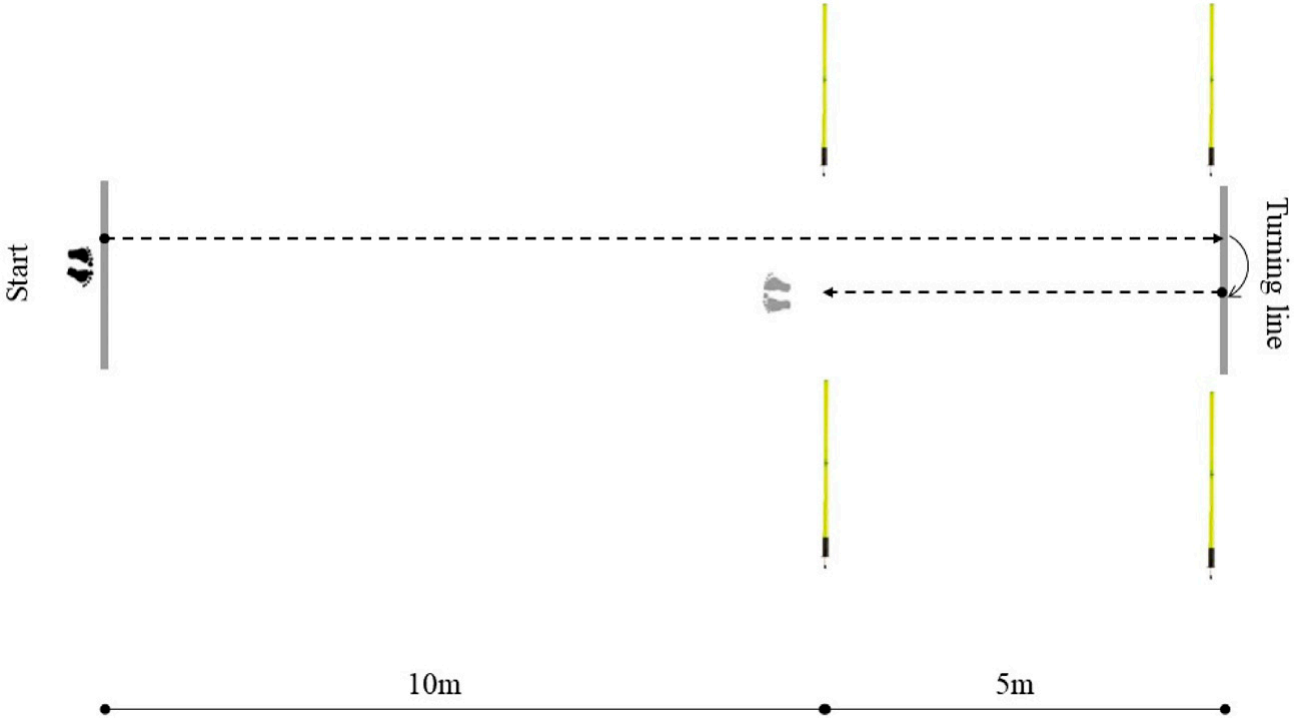
Layout of the 505 test performed with and without dribbling.

The 505 COD test was conducted following the established protocol outlined by Nimphius et al. (2016). Specifically, participants initiated a maximal effort sprint from a standing start position, 0.3 m behind the starting line. Participants sprinted through a timing gate towards a designated turning line marked on the hardwood floor. At the turning line, participants were instructed to place either their left or right foot on or behind the line before immediately sprinting back through the initial timing gate (Figure 3). Additionally, one researcher was positioned at the turning line to monitor the proper execution of the turns. Sprints were stopped and repeated if participants failed to adhere to the technical requirements, such as incorrect foot placement or premature change-of-direction. The 505 test is also one of the tests frequently used to assess change-of-direction ability in handball players (Fernandez-Fernandez et al., 2020; Ruscello et al., 2021). 505 test proved to be reliable (ICC: 0.88; CV: 2.40) (Stewart et al., 2014).

### Dribbling deficit

Dribbling the ball adds additional difficulties while performing tasks, and this difficulty manifests as the additional time needed to complete linear or change-of-direction sprint tasks, which is referred to as Dribble Deficit (Scanlan et al., 2018b). Dribble Deficit was calculated as the difference between the best total time for the dribbling trial and the best total time for the corresponding non-dribbling trial in both linear sprint performance tests and change-of-direction tests (Scanlan et al., 2018b; Conte et al., 2020).

### Statistical analysis

The mean ± standard deviation, 95% Confidence Intervals (95% CI) and Kolmogorov-Smirnov test were calculated for each outcome. Pearson’s two-tailed correlation was used to calculate relationships between Dribble Deficit, linear sprint performance, and change-of-direction with and without the ball. Correlation strength was interpreted as: small (r = 0 to 0.3 or −0.3 to 0), moderate (r = 0.31 to 0.49 or −0.31 to −0.49), large (r = 0.5 to 0.69 or −0.5 to −0.69), very large (r = 0.7 to 0.89 or −0.7 to −0.89), and perfect correlation (r = 0.9 to 1 or -0.9 to −1). Statistical analysis was performed using the IBM SPSS statistics program (version 26.0; Inc., Chicago, IL, United States). Statistical significance was set at p ≤ 0.05.

## Results

Significant large to very large correlations were observed between linear sprinting performance and dribbling time in the 20m linear test (*r* = 0.782; *R* ^*2*^ = 0.61, p < 0.001), slalom (*r* = 0.756, p < 0.001), zig-zag (*r* = 0.878, p < 0.001), and 505 test with dribbling (*r* = 0.658 p < 0.05) (Table 1, Table 2).

**TABLE 1:** Mean and standard deviation performance times with 95% confidence intervals (CI) for straight-line and change of direction (COD) sprint performance tests with and without ball dribble and corresponding dribble deficits (DD) in professional female handball players (n = 11).

			95%CI
	Mean	SD	[Lower; Upper]
10 m (s)	2.05	0.10	[1.99; 2.11]
20 m (s)	3.47	0.18	[3.36; 3.58]
30 m (s)	4.84	0.20	[4.72; 4.96]
10 m with dribbling (s)	2.19	0.26	[2.04; 2.34]
20 m with dribbling (s)	3.73	0.33	[3.53; 3.93]
30 m with dribbling (s)	5.15	0.20	[5.03; 5.27]
Slalom (s)	6.79	0.42	[6.54; 7.04]
Slalom with dribbling (s)	7.32	0.50	[7.02; 7.62]
Zig-Zag (s)	5.87	0.27	[5.71; 6.03]
Zig-Zag with dribbling (s)	6.29	0.32	[6.10; 6.48]
505 test (s)	2.46	0.11	[2.39; 2.53]
505 test with dribbling (s)	2.62	0.10	[2.56; 2.68]
Dribble deficit 10m (s)	0.14	0.22	[0.01; 0.27]
Dribble deficit 20m (s)	0.25	0.22	[0.12; 0.38]
Dribble deficit 30m (s)	0.31	0.19	[0.20; 0.42]
Dribble deficit slalom (s)	0.53	0.33	[0.33; 0.73]
Dribble deficit Zig-Zag (s)	0.42	0.15	[0.33; 0.51]
Dribble deficit 505 test (s)	0.16	0.09	[0.11; 0.21]

SD, standard deviation; 95% CI, 95% confidence intervals.

**TABLE 2:** Pearson correlations for the straight-line and change of direction (COD) sprint performance tests with and without dribbling in professional female handball players (n = 11).

Straight-line tests				
		With dribbling		
		10 m	20 m	30 m
Sprint	10 m	0.551		
	20 m		0.782**	
	30 m			0.530

Change of direction tests				
		With dribbling		
		Slalom	Zig-Zag	505 test
Sprint	Slalom	0.756**		
	Zig-Zag		0.878**	
	505 test			0.658*

** Significant at the p ≤ 0.01; * Significant at the p ≤ 0.05.

The correlation results between the linear and COD dribble deficits and test outcomes are presented in Tables 3, 4. Specifically, a perfect positive correlation was identified between the 10m Dribble Deficit and 10m dribbling time (r = 0.931, R^2^ = 0.87, p < 0.001). Similarly, a very large positive correlation was found between the 20m Dribble Deficit and 20m dribbling time (r = 0.884, R^2^ = 0.78, p < 0.001). In contrast, no significant correlations were observed between the 10m dribble deficit and 10m sprint performance time (r = 0.209, R^2^ = 0.04, p = 0.537), or between the 10m dribbling time and 10m sprint performance time (r = 0.551, R^2^ = 0.30, p = 0.079). Additionally, a moderate but no significant correlation was found between the 20m sprint time and 20m dribble deficit (r = 0.403, R^2^ = 0.16, p = 0.219).

**TABLE 3:** Relationship between dribble deficit (DD) and straight-line sprint performance tests with and without dribbling in professional female handball players (n = 11).

	DD 10m		DD 20m		DD 30m	
	*r* (95%CI)	*r rating (R* ^*2*^ *)*	*r* (95%CI)	*r rating (R* ^*2*^ *)*	*r* (95%CI)	*r rating (R* ^*2*^ *)*
10 m	0.209 [-0.45, 0.72]	*Small (R* ^*2*^ *= 0.044)*				
20 m			0.403 [-0.26, 0.81]	*Moderate (R* ^*2*^ *= 0.162)*		
30 m					−0.512 [-0.85, 0.13]	*Moderate (R* ^*2*^ *= 0.262)*
10 m with dribbling	0.931** [0.75, 0.98]	*Perfect (R* ^*2*^ *= 0.867)*				
20 m with dribbling			0.884** [0.60, 0.97]	*Very large (R* ^*2*^ *= 0.781)*		
30 m with dribbling					0.457 [-0.20, 0.83]	*Moderate (R* ^*2*^ *= 0.209)*

DD, Dribble deficit; ** Significant at the p ≤ 0.01; * Significant at the p ≤ 0.05.

**TABLE 4:** Relationship between dribble deficit (DD) and change of direction (COD) sprint performance tests with and without dribbling in professional female handball players (n = 11).

	DD slalom		DD zig-zag		DD 505 test	
	*r* (95%CI)	*r rating (R* ^*2*^ *)*	*r* (95%CI)	*r rating (R* ^*2*^ *)*	*r* (95%CI)	*r rating (R* ^*2*^ *)*
Slalom	−0.140 [-0.68, 0.50]	*Small (R* ^*2*^ *= 0.02)*				
Zig-Zag			0.078 [-0.55, 0.65]	*Small (R* ^*2*^ *= 0.006)*		
505 test					−0.552 [-0.87, 0.07]	*Large (R* ^*2*^ *= 0.305)*
Slalom with dribbling	0.542 [-0.09, 0.86]	*Large (R* ^*2*^ *= 0.294)*				
Zig-Zag with dribbling			0.545 [-0.082, 0.86]	*Large (R* ^*2*^ *= 0.297)*		
505 test with dribbling					0.264 [-0.40, 0.75]	*Small (R* ^*2*^ *= 0.07)*

DD, Dribble deficit; ** Significant at the p ≤ 0.01; * Significant at the p ≤ 0.05.

Further analyses revealed no significant correlations between Dribble Deficits in the slalom test (*r* = 0.542; *R* ^*2*^ = 0.29, p > 0.05) and zig-zag test (*r* = 0.545; *R* ^*2*^ = 0.30, p > 0.05) and their respective dribbling times. Finally, large negative but no significant correlation was found between Dribble Deficit 505 test and 505 test sprint time (*r* = −0.552; *R* ^*2*^ = 0.31, p > 0.05).

## Discussion

The study aimed to determine the relationship between linear and change-of-direction sprinting performance with dribbling performance and Dribble Deficit in professional female handball players. A large to very large correlation was found between linear sprinting performance and dribbling performance as well as between change-of-direction sprinting performance and dribbling performance. In addition, the study showed a moderate to perfect correlation between linear dribbling performance and Dribble Deficit, as well as a large relationship between change-of-direction dribbling performance and Dribble Deficit. In contrast, a small to moderate, non-significant relationship was observed between linear sprinting performance and Dribble Deficit, while a small, non-significant correlation was observed between change-of-direction sprinting performance and Dribble Deficit.

Proper assessment of basic sprinting and dribbling performance is a useful tool for practitioners to identify areas of improvement and develop appropriate training programs or create long-term athlete development plans. Our results showed a strong correlation between linear sprinting performance and dribbling performance, suggesting that faster female handball players also tend to be faster when dribbling, which strongly limited the current approach of dribbling proficiency assessment because of the very large influence of sprinting performance on overall results. Most importantly, this means that faster female handball players will be superior and achieve better results in dribbling tests regardless of their technical abilities, which clearly question dribbling quality and efficiency assessment based on the traditional approach to measure the time required to complete the course while dribbling the ball. In contrast to our results, Stamenković et al. (2023) did not find a significant correlation (R^2^ = 0.08, p = 0.303) between sprinting and dribbling performance over a short 10 m distance in male handball players, which may be related to the difficulty of standardizing of the test procedure. For example, some players may pass the ball 5 m and run, while others may take three touches over the same distance, which strongly influences overall results. On the other hand, our results are comparable with previous basketball studies (Scanlan et al., 2018b; Conte et al., 2020; Ramirez-Campillo et al., 2021a) who observed a large to very large relationship between linear sprinting and dribbling performance in senior semi-professional and collegiate male basketball players. We are aware of the limitations of comparing results with different genders and sports disciplines; however, to the best of our knowledge, this is the first study addressing the relationship between sprinting performance, dribbling performance, and Dribble Deficit in female handball players, so comparisons with similar female handball studies are limited.

When considering the relationships between sprinting and dribbling performance during change-of-direction performance, our results showed a large to very large relationship, indicating a strong overlap in the physical capacities required for both tasks. Our findings are congruent with previous male handball (Stamenković et al., 2023) and basketball (Scanlan et al., 2018b; Conte et al., 2020; Ramirez-Campillo et al., 2021a) studies concluding that change-of-direction dribbling performance is also strongly determined by a player’s running and sprinting performance. This finding underscores the importance of agility and the ability to change direction quickly as critical components of dribbling performance in handball. The strong relationship between these variables also suggests that improving change-of-direction performance could have a direct and substantial impact on a player’s dribbling capabilities.

To identify players’ strengths and weaknesses, assessment methods that evaluate the physical and technical components of performance independently should be used (Ramirez-Campillo et al., 2021a). The relationship between linear sprinting performance (10, 20 and 30 m) and Dribble Deficit was found to be small to moderate and non-significant in our study, which confirmed previous findings that Dribble Deficit is able to negate the impact of sprinting performance in the assessment of dribbling performance and effectively isolate the technical contribution to movement time (Conte et al., 2020; Ramirez-Campillo et al., 2021a). Similar to our study, Stamenković et al. (2023) also found a non-significant correlation between linear sprinting performance over 20 and 30 m and Dribble Deficit, while acceleration distance (10 m sprint) showed a significantly large correlation between linear sprinting and Dribble Deficit. As previously described, one of the reasons might be the challenge of standardizing testing methods with a ball over such a short distance and ensuring that all participants had the same number of contacts with the ball. Compared with studies being conducted in basketball, where dribbling also involves the use of the upper limbs, our findings are very similar, and all strongly negate the impact of linear sprinting performance on Dribble Deficit. This indicates that linear sprinting performance alone is not a strong predictor of the additional time required to dribble while sprinting. It suggests that factors other than straight-line performance, such as ball control and coordination, play a more crucial role in determining Dribble Deficit.

The relationship between change-of-direction sprinting performance and Dribble Deficit was small and non-significant, suggesting that change-of-direction performance alone does not have a significant effect on the additional time required to perform dribbling tasks. This emphasizes the notion that dribbling requires more complex skills than just the ability to change direction quickly. These findings are completely in line with previous male handball (Stamenković et al., 2023), semi-professional (Scanlan et al., 2018b), collegiate (Ramirez-Campillo et al., 2021a), and youth (Conte et al., 2020) basketball studies. Overall, this highlights that players who are faster at dribbling tend to have a lower Dribble Deficit, suggesting that efficient dribblers are less affected by the presence of the ball during sprinting.

Despite this study being the first to precisely show the impact of linear and change of direction sprinting performance on dribbling performance and Dribble Deficit in female handball players, there are several limitations. First, the relatively small sample size (n = 11) limits the generalizability of the findings to a wider population of handball players. Consequently, it was not possible to evaluate potential position-specific differences in Dribble Deficit. While all handball players require proficient dribbling skills, the specific demands of different positions (e.g., goalkeepers, wing players, line players) may vary. Thus, to further validate Dribble Deficit as a performance predictor, future studies should utilize a larger sample size to examine its relevance across various positions and its potential to differentiate performance in specific roles. Second, the study focused exclusively on senior, professional female handball players with well-established technical skills. This limits our understanding of Dribble Deficit in young handball players, where it may have significant implications for talent identification and training interventions. Third, the use of handball non-specific field tests, while allowing for comparison with previous research in team sports, may not fully capture the nuances of handball-specific movement patterns. Fourth, the study did not differentiate between dominant and non-dominant hand dribbling performance. More precisely, examining these differences could provide valuable insights into the underlying mechanisms of Dribble Deficit and inform specific training strategies to improve hand dominance and overall dribbling proficiency. Lastly, while our study utilized standardized tests to assess pre-planned changes of direction, we failed to examine the application of the Dribble Deficit in more complex movements and real-game situations. Given that reactive agility and cognitive abilities may influence Dribble Deficit, future research should investigate this construct within the context of these additional factors.

## Practical application

This study’s findings have significant implications for coaches, sports practitioners, and strength and conditioning specialists working with female handball players. Although this is the first study to investigate the dribbling deficit in professional female handball players, the results strongly suggest the utility of Dribble Deficit for assessing dribbling performance. Specifically, while handball players, on average, execute fewer dribbles than players in other team sports (Chelly et al., 2011; Stamenković et al., 2023), dribbling remains a crucial skill that significantly contributes to handball performance. Therefore, it is essential to evaluate dribbling separately, considering sprint performance and change-of-direction factors, since traditional approaches (e.g., total dribbling performance time) may not be the best option. Moreover, this study did not assess differences in Dribbling Deficit between playing positions, but coaches and practitioners should consider potential variations in performance when monitoring their players. Players with a high Dribble Deficit should prioritize specific skill-specific training to reduce this gap. Additionally, dribbling performance is often used in handball to identify talent and guide early development. Therefore, talent identification programs may benefit from incorporating Dribble Deficit as a key performance indicator, as it can help identify players with exceptional dribbling abilities, even if they may not possess superior sprinting performance.

## Conslusion

In conclusion, our study shows a strong correlation between different measures of performance (linear and change-of-direction) and dribbling performance in female handball players. While linear and change-of-direction performances are important, dribbling efficiency, particularly in change-of-direction scenarios, plays a crucial role in minimizing Dribble Deficit. Future research should further investigate these relationships, focusing particularly on the technical and skill-based aspects of dribbling, to develop targeted training interventions that can enhance overall performance in female handball players. In addition, longitudinal studies investigating how improvements in these variables affect match performance would provide deeper insights into the practical applications of our findings. Finally, future research should explore the influence of cognitive factors like decision-making and situational awareness on dribbling performance in real-game scenarios to further refine training strategies and performance enhancement.

## Data Availability

The raw data supporting the conclusion of this article will be made available by corresponding author upon reasonable request.
